# Supplementary material for: Causal Relationship between Aging and Anorexia Nervosa: A White-Matter-Microstructure-Mediated Mendelian Randomization Analysis
Source: Biomedicines. 2024 Aug 16;12(8):1874. doi: 10.3390/biomedicines12081874 (PMC11351342; doi:10.3390/biomedicines12081874)
Supplement: Supplementary file 1 [file biomedicines-12-01874-s001.zip › Supplementary Figure/Figure S4/Singe SNP plot of MR analysis for TL to ubm-b-2066.pdf]

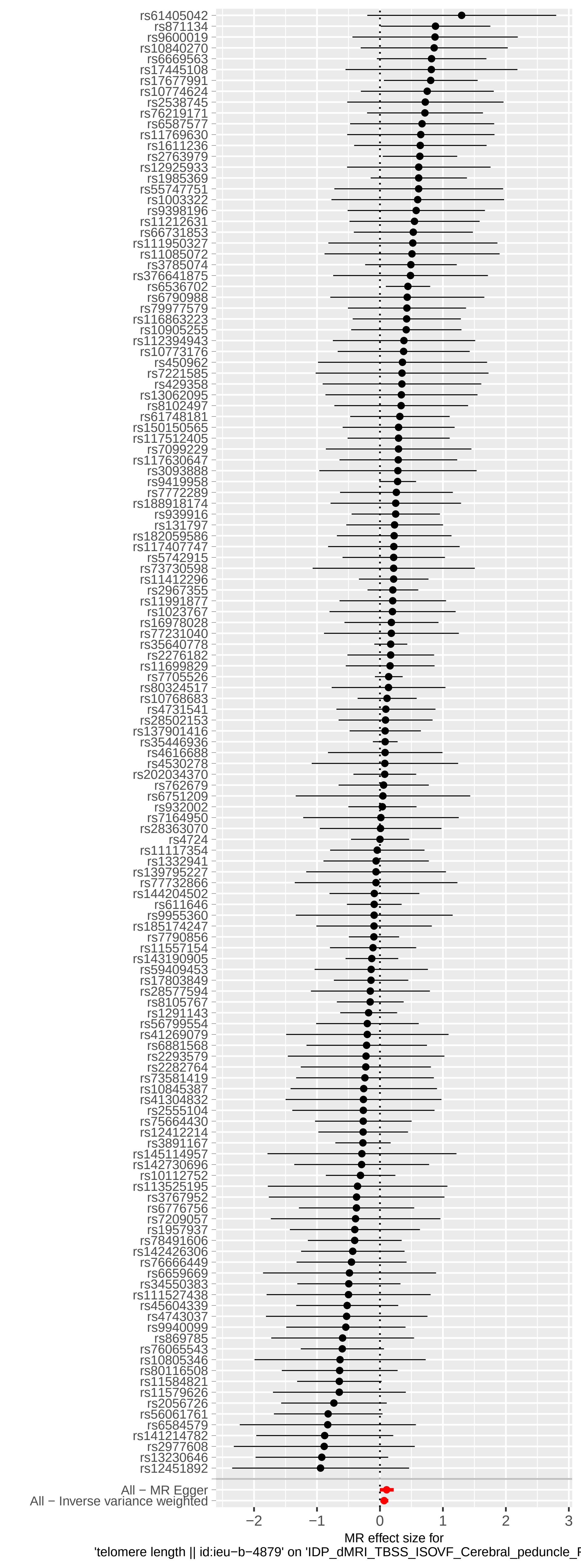
rs61405042
rs871134
rs9600019
rs10840270
rs6669563
rs17445108
rs17677991
rs10774624
rs2538745
rs76219171
rs6587577
rs11769630
rs1611236
rs2763979
rs12925933
rs1985369
rs55747751
rs1003322
rs9398196
rs11212631
rs66731853
rs111950327
rs11085072
rs3785074
rs376641875
rs6536702
rs6790988
rs79977579
rs116863223
rs10905255
rs112394943
rs10773176
rs450962
rs7221585
rs429358
rs13062095
rs8102497
rs61748181
rs150150565
rs117512405
rs7099229
rs117630647
rs3093888
rs9419958
rs7772289
rs188918174
rs939916
rs131797
rs182059586
rs117407747
rs5742915
rs73730598
rs11412296
rs2967355
rs11991877
rs1023767
rs16978028
rs77231040
rs35640778
rs2276182
rs11699829
rs7705526
rs80324517
rs10768683
rs4731541
rs28502153
rs137901416
rs35446936
rs4616688
rs4530278
rs202034370
rs762679
rs6751209
rs932002
rs7164950
rs28363070
rs4724
rs11117354
rs1332941
rs139795227
rs77732866
rs144204502
rs611646
rs9955360
rs185174247
rs7790856
rs11557154
rs143190905
rs59409453
rs17803849
rs28577594
rs8105767
rs1291143
rs56799554
rs41269079
rs6881568
rs2293579
rs2282764
rs73581419
rs10845387
rs41304832
rs2555104
rs75664430
rs12412214
rs3891167
rs145114957
rs142730696
rs10112752
rs113525195
rs3767952
rs6776756
rs7209057
rs1957937
rs78491606
rs142426306
rs76666449
rs6659669
rs34550383
rs111527438
rs45604339
rs4743037
rs9940099
rs869785
rs76065543
rs10805346
rs80116508
rs11584821
rs11579626
rs2056726
rs56061761
rs6584579
rs141214782
rs2977608
rs13230646
rs12451892
All − MR Egger
All − Inverse variance weighted
−2
−1
0
1
2
3
MR effect size for
'telomere length || id:ieu−b−4879' on 'IDP_dMRI_TBSS_ISOVF_Cerebral_peduncle_F
